# 

**Published:** 1915-02-06

**Authors:** 


### CONTENTS.

| | PAGE |
|---|---|
| **The Medical Lessons of the War** ... ... | 409 |
| **Hospital Experience and Higher Appointments** ... ... ... ... ... ... | 410 |
| **Hospital and Institutional News** ... ... ... | 411 |

The Poor-Law Conference—Should Conference Delegates be Paid their Expenses? — The Hampstead Appointment—Doctors' Commissions and Posts Kept Open—Remarkable Hospital Chapels—The Scarcity of Scientific Glassware—Motor Bacteriological Laboratory for the Welsh Army Corps—A Cottage Hospital for Coalville—The Art of Collecting—The Height of the Staff—Organising an Appeal Department — County Radium Institutes—Leeches from Australia—County Councils and the Public Health—Sanatorium Benefit Committee—The War and the School Medical Service—A Wise Decision—The Belvidere Hospital, Glasgow, Inquiry—A Journalist of the Old School—Orthopædics and the Wounded—Chester Infirmary's Finances—The Chadwick Lecture—Activities of Public Dispensers—Home-made Furniture : A Guinea Prize—Army Dispensing Again Disappointing—Gifts of Food from the Colonies—This Week's Drug Market.

| | PAGE |
|---|---|
| **War and Disease** ... ... ... ... ... | 417 |
| III.—The Great European War. | |
| **Smallpox and Vaccination in Siam** ... ... | 419 |
| A Remarkable Educational Campaign. | |
| **Round the World** ... ... ... ... ... | 420 |
| Fellow-Workers and the Roll of Honour. | |
| **Statistics and their Critics** ... ... ... ... | 421 |
| King Edward's Hospital Fund for London Statistics—Fourth Notice. | |
| **National Insurance Act Developments** ... ... | 423 |
| Report of Departmental Committee on Sickness Benefit Claims. | |
| **The Professional Life of London** ... ... | 425 |
| How it is Reacting to the War. | |
| **II.—A Visit to Stockholm Hospitals** ... ... | 426 |
| By a Travelling R.M.O. | |

[*See over.*]

### CONTENTS—(*continued*).

| | PAGE |
|---|---|
| **Rearrangements by the Metropolitan Asylums Board** ... ... ... ... ... ... ... | 426 |
| **The Editor's Letter-Box** ... ... ... ... | 427 |
| The Hampstead Appointment. | |
| Clinical Value of Poor-Law Infirmaries. | |
| **Public Pharmacists' Annual Meeting** ... ... | 428 |
| **Institutional Needs** ... ... ... ... ... | 428 |
| **The Institutional Library** ... ... ... ... | 430 |
| Mothercraft and Health Visitors. | |
| The "Legal" Engagement and Case Book. | |
| **The Institutional Worker** ... ... (Suppl.) | 1 |
| Answers to January Question: Receiving Goods Without a Steward. | |
| Decentralisation and Hints for Efficiency. | |
| Radiography and Some of its Difficulties. | |
| Question for February. | |
| Questions Invited. | |
| The Sick and in Need. | |
| Employment and Training. | |
| War Matters. | |
| Miscellaneous. | |

